# Charles-Jules-Henri Nicolle

**DOI:** 10.3201/eid1509.090891

**Published:** 2009-09

**Authors:** Myron G. Schultz, David M. Morens

**Affiliations:** Centers for Disease Control and Prevention, Atlanta, Georgia, USA (M.G. Schultz); National Institutes of Health, Bethesda, Maryland, USA (D.M. Morens)

Shown in this photograph is Charles-Jules-Henri Nicolle (1866–1936), a physician, microbiologist, novelist, philosopher, and historian. From 1903 until his death in 1936, he was Director of the Institut Pasteur in Tunis, Tunisia. Nicolle’s many accomplishments include the discovery that epidemic typhus is transmitted by body lice (*Pediculus humanis corporis*), discovery of the phenomenon of inapparent infection, and possibly the first isolation of human influenza virus after experimental transmission. Nicolle made many other fundamental contributions to knowledge of infectious diseases. This year is the centenary of his discovery about typhus transmission, made in the summer of 1909, for which he was awarded the 1928 Nobel Prize in Physiology or Medicine.

Nicolle was born on September 21, 1866, in Rouen, the ancient capital of Normandy, France. He obtained a classical education and was greatly attracted to literature, history, and the arts, interests he nurtured throughout his life. Bowing to the wish of his physician father, however, Nicolle studied medicine. After 3 years at the medical school in Rouen, he proceeded to Paris for further training and received a medical degree from the Institut Pasteur in 1893. At 27 years of age, Nicolle returned to his hometown, where he served as a member of the medical faculty and as Director of the Bacteriological Laboratory at L’École préparatoire de médecine et pharmacie de Rouen. His 8 years in Rouen were difficult: his position was untenured, his colleagues were reluctant to accept his modern ideas about bacteriology, and he experienced a hearing loss that prevented him from effectively using a stethoscope. These challenges may have motivated him to take a leap that he might otherwise not have taken when the post of directorship of the Institut Pasteur in Tunis became open. It was offered to his elder brother, Maurice (1862–1932), an established experimental scientist, who refused it. Charles then applied and obtained the position.

Nicolle arrived in Tunis in 1902, when he was 36 years old. North Africa was a good place to study infectious diseases, including brucellosis, diphtheria, leishmaniasis, leprosy, malaria, measles, Mediterranean spotted fever, relapsing fever, scarlet fever, tuberculosis, and typhus. Of all the problems Nicolle faced in Tunis, however, epidemic typhus was, in his words, “the most important and the least explored.” He studied it for the next 7 years. He was well aware of the clinical presentation of typhus—its triad of fever, rash, and stupor—and of its link to poverty. Throughout history, typhus had been a highly communicable and frequently fatal disease. Before it began to be understood as a single infectious disease distinguished epidemiologically from typhoid (in the mid to late 19th century), typhus had been considered a collection of distinctive diseases that affected specific populations. It devastated armies during wars (“war typhus”) and prisoners living under unsanitary conditions (“jail typhus” or “jail fever”); it affected displaced populations suffering from famine, floods, and other natural disasters; and in general, it was a disease of poverty.

In Tunis, typhus struck in seasonal waves during the cooler months and disappeared during the summer. It spread through overcrowded prisons, asylums, and tent villages, taking a heavy toll in hospitals among admissions personnel and sometimes even among examining physicians. Most of the doctors in the Tunisian health system, especially those in rural districts, had contracted typhus; approximately one third of them died from it. Nicolle’s first encounter with typhus could have potentially been his last. In 1903, he escaped death when at the last moment he cancelled a trip to investigate a prison outbreak. His 2 colleagues went on to the prison without him and spent the night there; both became ill with typhus and died.

Nicolle’s discovery of how typhus is transmitted came from observations at the entrance and waiting room of the Sadiki Hospital, which primarily served indigent patients. He often had to step over the bodies of typhus-infected patients who had fallen and died at the doorway. Nicolle observed that typhus patients who were admitted spread their infections to others up to the point at which they entered the hospital waiting room. Included among these secondary cases were persons who took charge of their clothing. However, patients became completely noninfectious as soon as they were bathed and dressed in a hospital uniform. They could then enter the general wards without posing a risk to others. Once Nicolle realized this, he reasoned that lice on patients’ clothes were most likely the vectors.

To test his hypothesis about lice, Nicolle requested and promptly received a chimpanzee (*Pan troglodytes*) from his mentor, Pierre-Paul-Émile Roux (1853–1933), at the Paris Institut Pasteur. Nicolle injected the chimpanzee with blood from a typhus patient. Twenty-four hours later, the chimpanzee was febrile, had new skin eruptions, and was prostrate. Because chimpanzees were costly, Nicolle then injected a toque macaque (*Macaca sinica*) with blood from the ill chimpanzee. Thirteen days later the macaque became febrile. Nicolle fed 29 lice on the ill macaque, and over the next few days transferred the lice to feed on other macaques. Eventually, macaques in this latter group became ill as well.

Thus, in June 1909, Nicolle reproduced typhus in a chimpanzee; in August 1909, he demonstrated that lice are the carriers of typhus; and in September 1909, he communicated his discovery to the French Académie des sciences. In these simple experiments, Charles Nicolle had solved the mystery surrounding the transmission of one of humankind’s most dreaded scourges, a disease that had been a major force in shaping world history. Later research showed that the principal transmission method was not the bites of lice but the excrement of lice rubbed into the skin or eyes.

Hans Zinsser (1878–1940), an American microbiologist and historian, dedicated his classical work, Rats, Lice and History, to Charles Nicolle “with affectionate friendship.” In his autobiography, Zinsser speaks of Nicolle’s qualities as a scientist:

Nicolle was one of those men who achieve their success by long preliminary thought, before an experiment is formulated, rather than by the frantic and often ill-conceived experimental activities that keep lesser men in ant-like agitation... Nicolle did relatively few and simple experiments. But every time he did one, it was the result of long hours of intellectual incubation during which all possible variants had been considered and were allowed for in the final tests. Then he went to the point, without wasted motion … In the case of the louse discovery, Nicolle had carried out no more than a half-dozen decisive experiments after years of observation of the disease and its epidemiology. In this instance, his experiments were easily confirmed.

Indeed, in the year after Nicolle’s typhus discovery, Howard Taylor Ricketts (1871–1910) and Russell Morse Wilder (1885–1959), working in Mexico, confirmed louse transmission of typhus. In 1916, Henrique da Rocha-Lima (1879–1956) identified the causative organism and named it *Rickettsia prowazeki* in memory of Ricketts and Stanislaus Joseph Matthias von Prowazek (1875–1915), both of whom had died of typhus contracted during their scientific investigations.

Although Nicolle is not credited with discovering the cause of human influenza, his contributions were seminal. In 1903, when he had just joined the Institut Pasteur in Tunisia, his mentor Émile Roux reviewed the literature on “filter-passing” agents (hypothetical subbacterial agents that passed through Berkfeld and Chamberland filters). Roux identified 10 of them that he believed to be scientifically proven as causative agents of disease, among them what we now know to be viruses and mycoplasmas. Working at Turkey’s Imperial Institute of Bacteriology, Nicolle’s brother Maurice and colleagues had isolated the filter-passing agent of rinderpest (later characterized as a paramyxovirus). Charles Nicolle, who had also worked with rinderpest, was familiar with these new techniques.

When the deadly influenza pandemic struck in 1918, Nicolle was among the few scientists in the world prepared to study its etiology. At the time, the cause of influenza was unknown, but many doubted the conventional explanation that it was a bacterial disease. Beginning on September 1, 1918, Nicolle injected Chamberland-filtered and unfiltered sputum samples from ill patients into human volunteers and into monkeys, reproducing in some experiments a febrile influenza-like illness. However, the scarcity of clinical material and the rapidity with which the epidemic advanced precluded large-scale controlled studies. Within a few months, a Japanese group appeared to reproduce and extend the results of the 2 French scientists, but other investigators had trouble doing so. As the pandemic faded into endemicity, further experimentation became difficult for all researchers. When influenza viruses were eventually isolated and characterized in mice and in ferrets more than a decade later, Nicolle was finally acknowledged as having made the first isolation and as having taken the first important steps toward finding influenza’s cause.

In addition to increasing knowledge about typhus and influenza, Nicolle made important contributions to the understanding of brucellosis, leishmaniasis, measles, rinderpest, scarlet fever, Mediterranean spotted fever, toxoplasmosis, trachoma, and tuberculosis. Perhaps his greatest discovery, a critical key to understanding the epidemiology of many infectious diseases, was characterization of the phenomenon of inapparent infection, the acquisition and transmission of infection without signs of illness. This line of work began with Nicolle’s observations on experimental typhus. He learned that guinea pigs were good hosts for the typhus organism and showed that certain guinea pigs could have apyretic typhus after a primary infection of pyretic typhus. Nicolle extended his observation to other infections—viral, bacterial, and parasitic—finding similar phenomena in each. As Charles-Edward Amory Winslow (1877–1957) emphasized in his classical work, The Conquest of Epidemic Diseases: A Chapter in the History of Ideas (1943), inapparent infection is one of the most important concepts in infectious disease epidemiology, and it had for centuries been one of the key missing links, which prevented full understanding of the principles of disease transmission. Inapparent infection of symptomless carriers is now generally accepted as the source for dissemination of many communicable diseases. Nicolle considered it his most important discovery.

Nicolle wrote several philosophical works, including Destin des maladies infectieuses (1933); La nature; conception et morale biologiques (1934); Responsabilités de la médecine (1936); and La destinée humaine (1941). Nicolle also wrote fanciful stories, such as Le pâtissier de Bellone (1913), Les deux larrons (1929), and Les contes de Marmouse et de ses hôtes (1930). His novels brought him great pleasure and a circle of admiring readers.

Nicolle’s discovery of the means of transmission of typhus can be viewed as both a beginning and an end. It ended a 20-year epoch in which arthropods were found to be the vectors of major diseases of animals and humans, including not only typhus but also African trypanosomiasis, American trypanosomiasis, dengue, filariasis, malaria, relapsing fever, Texas cattle fever, and yellow fever. This epoch was as successful in the history of medicine as had been the phenomenal development of bacteriology in the decades immediately preceding it. Nicolle’s discovery was also the beginning of the end of epidemic typhus. In the late 1930s, Paul Müller (1899–1965) discovered that dichlorodiphenyltrichloroethane (DDT) was highly effective for killing lice and other insects. During World War II, several potentially severe epidemics of typhus, especially the epidemic in Naples, Italy, in 1943–1944, were averted by dusting at-risk populations with DDT. Epidemic typhus had reigned for centuries, extinguishing millions of lives prematurely. Now it is an uncommon epidemic disease. The combined discoveries of Nicolle and Müller are compelling proof of the melioristic notion that the world becomes a better place through sustained human effort. Charles Nicolle, a modest Renaissance man who toiled in Africa, far from the scientific limelight of Berlin, New York, and Paris, is remembered for forever changing biomedical science and for having contributed to saving the lives of millions.
